# Cyclophosphamide and the taste system: Effects of dose fractionation and amifostine on taste cell renewal

**DOI:** 10.1371/journal.pone.0214890

**Published:** 2019-04-04

**Authors:** Eugene R. Delay, Sarah H. Socia, Jessica L. Girardin, Benjamin C. Jewkes, John H. King, Rona J. Delay

**Affiliations:** Department of Biology and Neuroscience Program, University of Vermont, Burlington, Vermont, United States of America; University of Modena and Reggio Emilia, ITALY

## Abstract

Chemotherapy often causes side effects that include disturbances in taste functions. Cyclophosphamide (CYP) is a chemotherapy drug that, after a single dose, elevates murine taste thresholds at times related to drug-induced losses of taste sensory cells and disruptions of proliferating cells that renew taste sensory cells. Pretreatment with amifostine can protect the taste system from many of these effects. This study compared the effects of a single dose (75 mg/kg) of CYP with effects generated by fractionated dosing of CYP (5 doses of 15 mg/kg), a dosing approach often used during chemotherapy, on the taste system of mice using immunohistochemistry. Dose fractionation prolonged the suppressive effects of CYP on cell proliferation responsible for renewal of taste sensory cells. Fractionation also reduced the total number of cells and the proportion of Type II cells within taste buds. The post-injection time of these losses coincided with the life span of Type I and II taste cells combined with lack of replacement cells. Fractionated dosing also decreased Type III cells more than a single dose, but loss of these cells may be due to factors related to the general health and/or cell renewal of taste buds rather than the life span of Type III cells. In general, pretreatment with amifostine appeared to protect taste cell renewal and the population of cells within taste buds from the cytotoxic effects of CYP with few observable adverse effects due to repeated administration. These findings may have important implications for patients undergoing chemotherapy.

## Introduction

Patients undergoing chemotherapy often report that their sense of taste has been adversely affected by their treatment [1, 2]. This usually involves a loss of sensitivity for one or more basic tastes but can also manifest as dysgeusia or as hypersensitivity [3–6]. In chemotherapy patients, disturbances in taste can have a negative impact on nutritional intake, reduce energy intake at a time when an increase in energy is necessary, and ultimately result in a poorer clinical prognosis [7–10]. Frequently, the clinical approach to chemotherapy is to divide the therapeutic dose into parts to be administered over time, an approach often called dose fractionation [11–13]. Fractionated dosing assumes that rapidly proliferating cancerous cells are exposed to the chemotherapy drug over a longer period to increase its effectiveness. Because each dose is smaller than the full dose, side effects may be lessened or eliminated. By extension, however, one would expect normal tissues requiring rapid cell renewal to be adversely affected by dose fractionation. The taste system is known for the short life spans of taste sensory cells and relatively rapid cell renewal of these cells. One goal of this study was to determine if dose fractionation of a chemotherapy drug, cyclophosphamide (CYP), might change the way the drug affected the taste system.

CYP, one of the earliest of the chemotherapy drugs still used today for treating certain types of cancers, is a prodrug that is converted into acrolein and phosphoramide mustard by the P450 system [14]. While both metabolites are cytotoxic, phosphoramide mustard is an alkylating agent that targets open DNA [15]. This makes CYP particularly toxic to cells engaged in cell renewal, such as cancerous cells or normal cells with short life spans requiring frequent renewal. Previous research with mice has shown that CYP can disrupt taste functions by increasing taste thresholds and decreasing the ability to discriminate different tastes [16–19], killing taste sensory cells within taste buds, and suppressing cell renewal involved in replacement of aging taste sensory cells [16, 19]. Several types of cells are found within a taste bud [20, 21]. Type I cells, the most common cell type, are glial-like and appear to support the health of other cells within the taste bud. Type II cells detect sweet, bitter and umami taste substances with receptors coupled to phospholipase C (PLC) second messenger systems and use ATP as a neurotransmitter [22–25]. Type III cells detect sour substances and form classical synaptic connections with afferent neurons [21, 26–28]. Type I cells were the first to be implicated in salt taste, but it now appears that all three cell types may contribute to salt taste [21, 29]. Each of these cell types appears to have a limited life span [30]. Type II cells have a half-life of 6–8 days, Type III cells have half-lives of about 22–24 days, and Type I cells appear to have half-lives of 6–8 days, although a subset may live much longer. The short life spans of these cell types require constant replacement by proliferating cells in a basal layer just below taste buds that maintain the working population of cells within taste buds. These progenitor cells produce daughter cells which, if they enter into a taste bud and transiently express *Sonic Hedgehog*, are considered Type IV cells that are immediate precursor cells to the other three cell types [31–34]. A recent comprehensive study showed that the Wnt/β-catenin pathway plays a major role in regulation of taste cell proliferation and taste cell renewal processes [35]. The rate of cell cycling of the proliferating cells is relatively high [31], making these cells especially vulnerable to the effects of CYP. A single dose of 75 mg/kg can kill cells within taste buds and severely depress the rate of cell cycling within the basal layer for several days [16, 19]. When mature cells die and there are inadequate replacement cells, additional disruptions in behavioral taste functions occur [16, 18].

The half-life of CYP is less than 6 hours and is generally eliminated from the system within 24 hours [36] but its impact on the taste system lasts for days after treatment. The time course of the effects of a single 75 mg/kg dose of CYP on the taste system of mice has been well delineated by behavioral measures of sucrose and umami thresholds evaluated daily for over two weeks post injection [16, 18]. A recent study with mice also reported elevated thresholds for NaCl that peaked eight days after a single dose (100 mg/kg) of CYP [37]. However, after receiving fractionated doses of CYP (20 mg/kg) daily for five consecutive days, increases in NaCl thresholds were longer lasting and more cyclic than observed after the single large dose. This suggests that dose fractionation of CYP may have somewhat different and more potent effects on cells within taste buds and possibly cell renewal than a single large dose of CYP.

Much of the impact of CYP on the taste system can be alleviated by pre-treatment with amifostine (AMF) [16, 17], a drug used clinically as a pretreatment to irradiation or chemotherapy [38]. AMF can enter normal tissues where it can scavenge free oxygen radicals but cannot enter cancer cells that have a lower pH [39, 40]. Healthy cells also have higher concentrations of the enzyme alkaline phosphate, which is necessary to metabolize AMF into its active component, WR-1065. WR-1065 can stabilize genes regulating DNA repair and cell cycle activity, thereby helping normal cells survive the effects of chemotherapy or radiotherapy [41, 42]. Mukherjee et al. [16, 17] reported that 100 mg/kg AMF given to mice subcutaneously 30 minutes prior to 75 mg/kg CYP reduced cell loss within the taste system and reduced elevations in sucrose thresholds initially observed 2–4 days after CYP injection and again 6–14 days after injection when the largest increase occurred. AMF appears to be tolerated well by most patients [38], but it can also have detrimental effects by itself [43–47]. Mukherjee et al. found that, while AMF was able to reduce the impact of CYP on the taste system, AMF also appeared to have mild detrimental effects on taste sensory cells after a single exposure [16, 17]. No one has yet examined the effects of repeated exposure to AMF on taste buds, however.

Two primary issues were addressed in this study using immunohistochemical assays. First, we asked whether dose fractionation of CYP had effects on the taste system comparable to those of a single large dose. Because fractionated dosing of CYP altered behavioral thresholds for salt differently than a single large dose, we wanted to compare the effects of two dosing regimens of CYP on populations of different cell types of murine circumvallate taste buds over days post injection. While the time course of the behavioral effects of CYP are well characterized, the effects of CYP on taste cells are not as well delineated since cellular effects have only been observed after a single dosing at 4 and 8, 10, 12 and 16 days post injection [16, 18, 19]. To address this question more accurately we examined taste buds at two-day intervals following either a single large dose of CYP or five smaller doses of CYP. Second, we asked whether AMF can protect the taste system similarly with either dosing regimen and whether repeated exposure to AMF would undermine some of its apparent protective effects. To address these two questions, we examined the effects of AMF and AMF+CYP at 4 and 10 days after the last injection of CYP and/or AMF.

In general, we found that fractionated dosing of CYP had more adverse and more prolonged effects on the taste system than single dosing. AMF appears to reduce or eliminate many of the effects of CYP on taste with few detrimental effects on the taste system after repeated dosing alone or as a pretreatment to CYP.

## Materials and methods

### Ethical consideration

This study followed strict accordance with recommendations in the Guide for the Care and Use of Laboratory Animals of the National Institutes of Health. All experimental procedures were reviewed and approved by the University of Vermont’s Institutional Animal Care and Use Committee (IACUC protocol: 14–003). All mice were euthanized by sodium pentobarbital (SleepAway, Zoetis Inc, Kalamazoo, MI, USA) overdose followed by transcardial perfusion. All efforts were made to minimize suffering.

### Animals

For these experiments six- to eight-week old male C57BL/6J mice were obtained from Jackson Laboratory (Stock No: 000664; https://www.jax.org/strain/000664; Bar Harbor, ME, USA) and habituated to the colony for at least seven days before random assignment to an experimental group. All mice were between 9–12 weeks of age at the beginning of the experiment and weighed between 23–32 g. Food (Purina Mouse Chow, Prolab RMH 3000) and water were available ad libitum. The mouse colony was maintained on a regular 12/12 hr light-dark cycle. To minimize the number of mice, whenever possible tissue sections from each mouse were used for processing with all three fluorescent markers in that experimental condition.

### Drug treatments

To select a base dose of CYP for these experiments, we conducted a preliminary experiment to examine the effects of four different doses (18.75, 37.5, 75 and 150 mg/kg body weight). At 75 and 150 mg/kg the effects of a single intraperitoneal (IP) injection of CYP were detectable in the taste system. However, CYP can cause nephrotoxicity at 150 mg/kg or greater [48–50]. On the other hand, the cellular effects of a single dose of 37.5 mg/kg were more difficult to detect and the effects of 18.75 mg/kg on circumvallate taste buds were marginally detectable. Therefore, 75 mg/kg was selected for this study. It also allowed us to relate the current findings to our initial behavioral experiments [16, 18]. All CYP solutions were prepared fresh in saline on the same day of administration.

#### Experiment 1: Dose fractionation

The first experiment was designed to compare effects of a single dose of CYP (Cyclophosphamide monohydrate, Acros Organics, New Jersey, USA) to a dose fractionation regime on the taste system of mice. Mice were randomly assigned to one of three drug treatment conditions. In the saline treatment condition (SAL), the mouse was given saline injections (IP, 1 ml/kg body weight) for five consecutive days. Mice in the second treatment condition received four consecutive days of saline injections and, on the fifth day, a dose of 75 mg/kg CYP, hereafter referred to as the 1CYP treatment condition. Mice in the third treatment condition received an injection of 15 mg/kg CYP each day for five consecutive days, hereafter referred to as the 5CYP treatment condition. To evaluate the effects of each treatment condition, mice were overdosed, perfused and tissues harvested at 2-day intervals up to 16 days post injection. A total of 111 mice were used in this experiment. The number of mice in each experimental group is listed in S1 Table. Injections were given at the same time each day. Mice were placed into new cages and bedding daily to minimize re-exposure to the metabolites of either drug that were excreted and absorbed by the bedding.

#### Experiment 2: AMF pre-treatment

This experiment was designed to address two questions: 1) can AMF protect the taste system from either CYP dose regime, and 2) does repeated exposure to AMF have a detrimental effect on the taste system? A previous study [16, 17] reported that when 100 mg/kg AMF is administered subcutaneously 30 min prior to CYP injection, it can protect the taste system from much of the damage induced by CYP. To evaluate the effects of AMF, mice were randomly assigned to one of the two dosing regimens used in experiment 1. Each of these groups was further divided into four drug groups (S2 Table): SAL only, CYP only, AMF only, or AMF+CYP. CYP mice received an injection daily for five days and were given either four injections of saline and the last injection of 75 mg/kg CYP, or five days of 15 mg/kg CYP. AMF groups received 100 mg/kg subcutaneously either once after four days of saline injections or daily for five consecutive days. AMF+CYP mice received 100 mg/kg AMF 30 min prior to receiving their assigned CYP dose. All SAL mice received five injections of saline and were randomly assigned to one of the two dosing regimens. To simplify the nomenclature, the groups will be identified by the number of CYP and/or AMF doses that they received combined with the drug treatment to which they were assigned: 1SAL, 5SAL, 1CYP, 5CYP, 1AMF, 5AMF, 1AMF+CYP, or 5AMF+CYP. In addition, initial data from experiment 1 indicated that both CYP dosing conditions significantly altered the taste system at 2–4 days and 8–10 days post injection. Therefore, each of the eight groups of mice was further divided to be overdosed and perfused 4 or 10 days post injection for tissue collection. These days were picked because they were time points when both dosing regimens had comparable disruptive effects on each type of taste cell. Day 4 disruptions appear to be related to the immediate effects of the drugs on the taste system whereas disruptions seen on day 10 appear to be related to secondary effects of the drugs. Thus, the overall design of this experiment involved 3 factors: 1) 2 dosing regimens, 2) 4 drug treatment conditions, and 3) 2 post injection intervals (4 and 10 days post injection). A total of 179 mice were used in this experiment. The number of mice in each experimental group is listed in S2 Table.

### Tissue collection

Following euthanasia, transcardial perfusion was performed using Heparin-1X in 0.1 M phosphate buffered saline (PBS) and 4% paraformaldehyde mixed in PBS. Tongues were dissected and fixed in the 4% paraformaldehyde solution for 3 hrs before blocking the tongue into three sections. The tissues were then incubated through a series of sucrose concentrations (0.5, 1.0 and 1.5 M) for cryo-protection before embedding in OCT compound (Tissue Tek, Sakura Finetec USA Inc., Torrence, CA) for storage at -80^o^ C. Tissue sections were cut at 12 μm thickness and mounted directly onto slides. Adjacent sections were mounted sequentially on 6 separate slides. This process was repeated until the entire circumvallate papilla was sectioned to ensure all slides had comparable tissue sampling and could be used for any of the fluorescent markers in that experiment. These slides were stored at -80^o^ C until processing. For these experiments, the focus was on circumvallate papilla containing taste buds along the walls of its crypt.

### Immunohistochemistry

#### Ki67 labeling

To compare the effects of drug treatment on cell proliferation, we labeled Ki67, a protein expressed by cells in all phases of cell cycle except G0 and early G1. For this procedure, slides were first washed in PBS, then bathed in 10 mM sodium citrate (pH 6.0) at 95^0^ C for 15 min to induce antigen retrieval before cooling to room temperature. After washes in PBS, the tissues were blocked with a mix of 5% NGS, 1% BSA and 0.3% Triton-X 100 for 1 hr at room temperature. The rabbit anti-Ki67 primary antibody (1:200 dilution; catalog # SP6, ThermoFisher Scientific; RRID: AB-2341197) was added to the tissues, then covered with hybristrips for incubation overnight at 4^0^ C. The following day sections were washed in PBS before they were incubated for two hrs in Alexa 546 goat-anti-rabbit secondary antibody (1:1000 dilution, Invitrogen, ThermoFisher Scientific, Waltham, MA, USA RRID: AB_2534093) at room temperature. Sections were then washed in PB before bathed in Sytox green (1:30,000, catalog # S7020, Molecular Probes, Eugene, OR, USA) to double label nuclei. After additional washes, cover slips were mounted using Fluoromount G (#0100–001, Southern Biotech, Burmingham, AL, USA).

#### PLCβ2-labeling

Immunomarkers for Type II and Type III cells were used to determine if differentiated, mature taste cells were affected by drug treatment conditions. More specifically, we used immunofluorescent markers for PLCβ2 to identify Type II cells and SNAP-25 to identify Type III cells. For PLCβ2 labeling, slides were washed in PBS and then incubated with 5% NGS in blocking solution for 1.5 hr at room temperature. The tissues were then incubated in rabbit anti-PLCβ2 primary antibody (H-255, Santa Cruz Biotechnology, Dallas, TX; RRID: 2163248) at 1:1000 dilution overnight at 4^0^ C. Alexa 546 goat-anti-rabbit secondary antibody (1:1000) was used to incubate the tissues for 2 hrs in the dark. Sytox green was used as a nuclear marker.

#### SNAP-25 labeling

The protocol for SNAP-25 was the same as for PLCβ2 (protocol courtesy of Dr. L. Barlow, University of Colorado School of Medicine, Aurora, CO). Tissues were incubated in rabbit anti-SNAP-25 primary antibody (1:5000; Sigma Aldrich cat # S9684; RRID: AB_261576) overnight at 4^0^ C. The concentrations, times of treatment of the Alexa 546 secondary antibody, and Sytox labeling procedures were the same as above.

### Image analysis and statistics

Immunofluorescent images were captured using a color camera (Spot RT KE Diagnostic Instruments Inc.) mounted on a Nikon Eclipse E600 Scope and Spot acquisition software (Spot Advanced, Version 5.1). Cell counts were done by observers blind to the experimental conditions using the criteria of Nguyen et al. [51]. Images were enhanced as needed to more clearly identify immuno-positive cells in Adobe Photoshop CS6 (https://www.Adobe.com) by adjusting brightness and RGB levels before quantification using NIH ImageJ (https://imagej.nih.gov/ij). The percentage score for Ki67 was calculated by dividing the number of immuno-positive cells for Ki67+ cells by the total number of basal epithelial cells or Sytox-positive taste sensory cells. Only cells within the basement layer and within the walls of the crypt of the circumvallate papilla that contained taste buds were counted. The data for PLCβ2 and SNAP-25 labeling were means of 5–12 taste buds per mouse. The number of immune-positive cells and the total number of nuclei labeled with Sytox green were counted within each taste bud. The data for all experiments were collected from 3 to 6 mice per group (S1 and S2 Tables).

Linear model analyses of variance (ANOVA) were used to analyze the data for Ki67, PLCβ2, and SNAP-25. Separate ANOVAs were performed to evaluate counts of labeled cells, total number of cells within taste buds, and the percentage of labeled cells within taste buds for each cellular label. For experiment 1 (dosing regimens), these ANOVAs examined dosing conditions (3 levels: SAL, 1CYP, and 5CYP) and days post treatment (8 levels: days 2–16) treated as between subject variables. To identify significant group differences, the data were then partitioned by day and by dosing factors to further identify group differences using ANOVA simple effects tests, and post hoc t-test with Sidak alpha correction procedures [52]. For experiment 2 (AMF pretreatment), 3-way ANOVAs were initially applied to the data for each dependent variable, then partitioned to identify significant differences between dosing conditions. All statistical tests were performed with SPSS version 24.0 (IBM Software, Chicago, IL). Covariance was treated as first order auto-regressive or AR(1), assuming no sphericity of the data. Graphs were made with GraphPad Prism 8 (GraphPad Software Inc., La Jolla, CA, USA).

## Results

### Experiment 1: Dosing regimens

**Fig 1:**
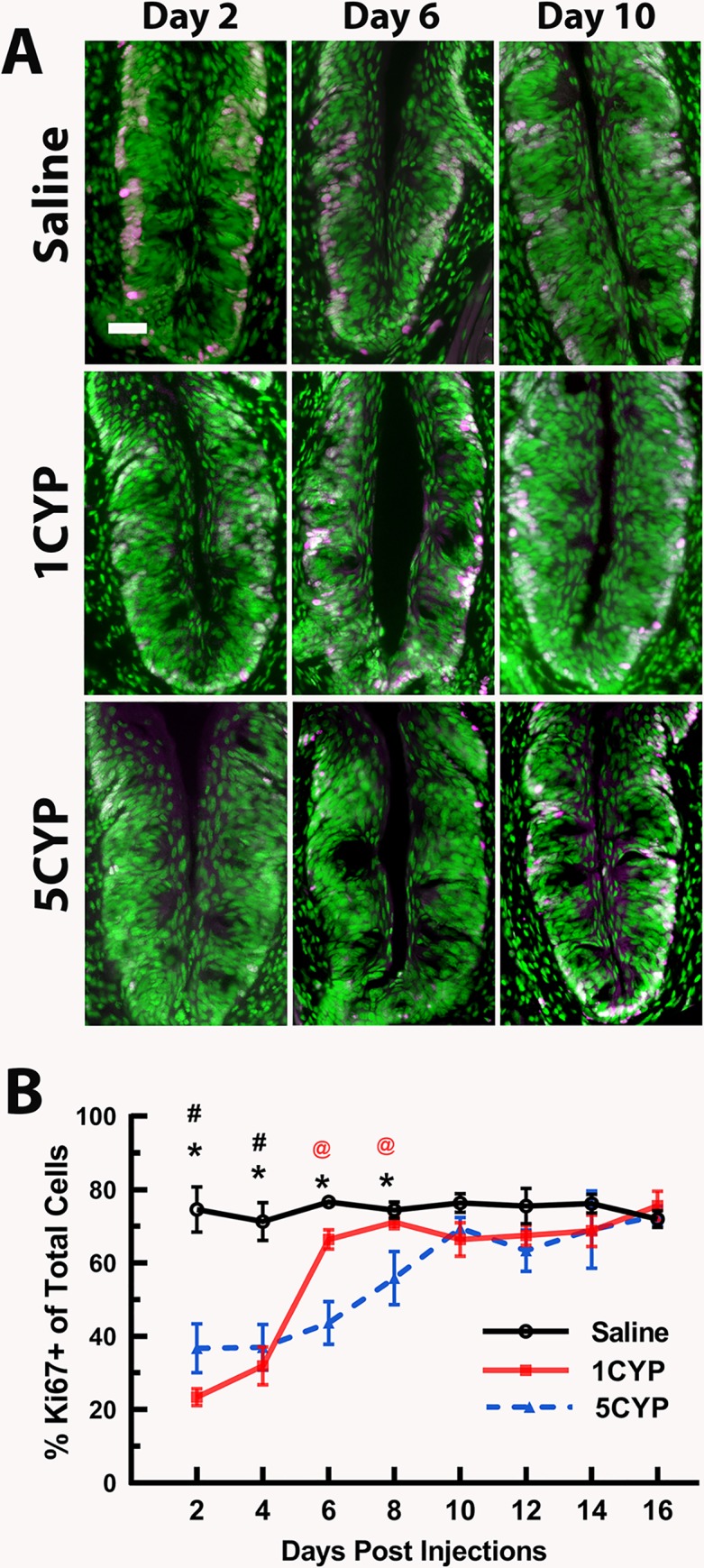
Cell proliferation in the basal layer of circumvallate papillae was differently reduced by the two CYP dosing regimens. (A). Ki67+ cells (magenta) in circumvallate papillae after saline injections (Saline, top row), a single injection of 75 mg/kg CYP (1CYP, middle row), or five injections of 15 mg/kg CYP (5CYP, bottom row) at 2 days (left column), 6 days (middle column), and 10 days (right column) post injection. Tissues were counter-reacted with Sytox green, a nuclear marker. For the 1CYP mice, Ki67+ cells were much reduced 2 days after injection. For the 5 CYP mice, Ki67 was suppressed at 2 and 6 days post injection. Scale bar = 20 μm. (B) Means ± SEM percent of Ki67+ cells relative to total cells plotted over days post injection. For 1CYP mice, Ki67 signal was significantly lower than saline controls at 2 and 4 days after CYP injection. For 5CYP mice, Ki67+ label was significantly lower than controls 2–8 days post injection and was significantly lower than 1CYP mice at 6 and 8 days post injection. All Ps < .05 or less: (1) * 5CYP < Saline, (2) # 1CYP < Saline, and (3) @ 5CYP < 1CYP.

**Fig 2:**
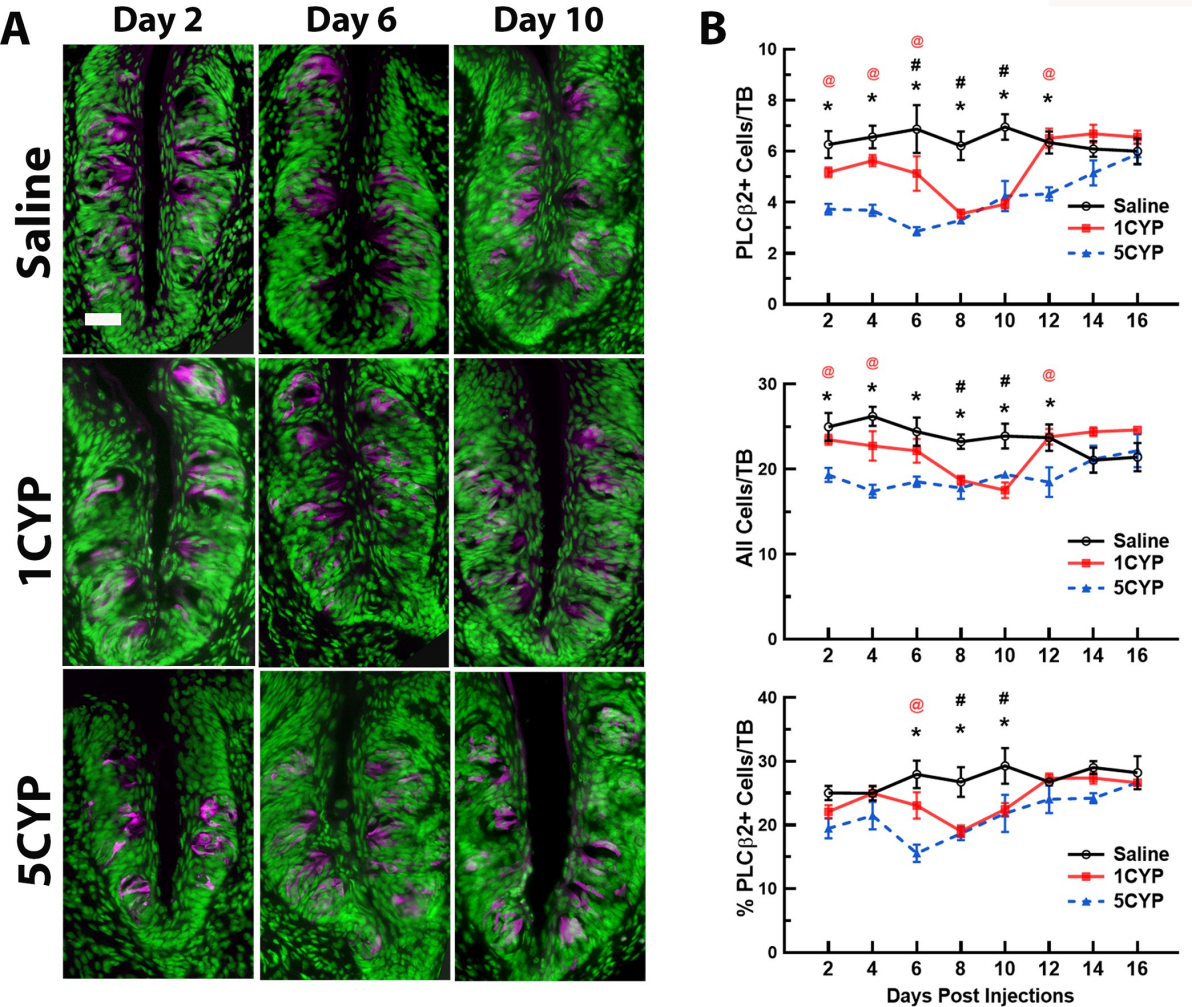
CYP dosing conditions differentially altered PLCβ2 expression in circumvallate taste buds. (A). PLCβ2+ cells (magenta) in taste buds after saline injections (Saline, top row), a single injection of 75 mg/kg CYP (1CYP, middle row), or five injections of 15 mg/kg CYP (5CYP, bottom row) at 2 days (left column), 6 days (middle column), and 10 days (right column) post injection. Tissues were counter-reacted with Sytox green, a nuclear marker. For the 1CYP mice, PLCβ2+ cells were much reduced 8–10 days after injection. For the 5CYP mice, there were fewer PLCβ2+ cells at 6–10 days post injection. Scale bar = 20 μm. (B) Means ± SEM of PLCβ2+ cells (top graph), total cells (middle graph), and percent of PLCβ2+ cells per taste bud (bottom graph) are plotted over days post injection. For 1CYP mice, PLCβ2+ signal began to decrease at 6 days and was significantly lower than controls at 8 and 10 days after CYP injection. For 5CYP mice, PLCβ2+ and total number of cells per taste bud were significantly lower than saline controls 2–12 days post injection and significantly less than 1CYP mice at 2–6 and 12 days post injection. The percent of PLCβ2-labeled cells in 5CYP mice were lower than 1CYP mice at 6 days and lower than controls days 6–10. All Ps < .05 or less: (1) * 5CYP < Saline, (2) # 1CYP < Saline, and (3) @ 5CYP < 1CYP.

**Fig 3:**
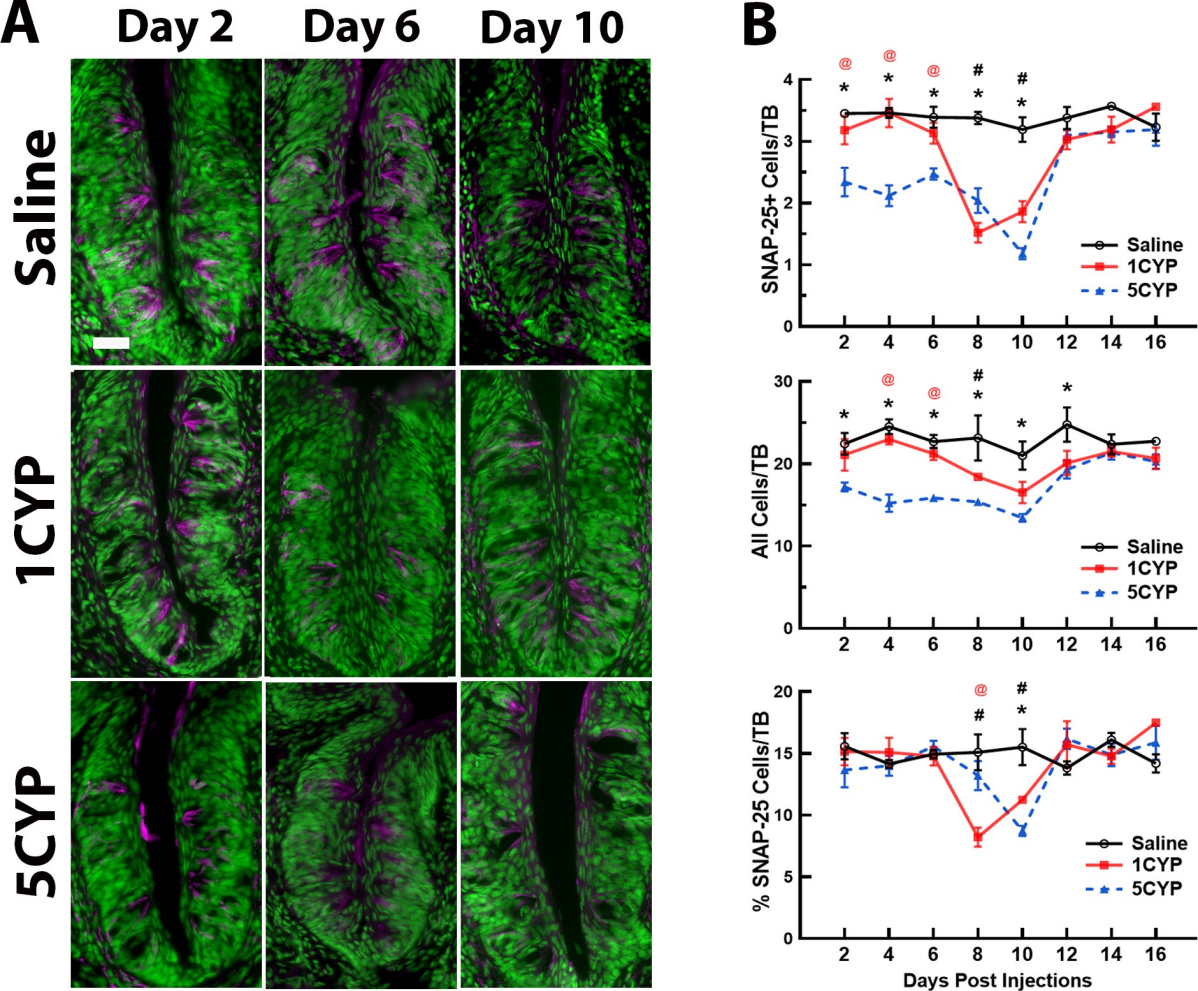
CYP dosing conditions differentially altered SNAP-25 expression in circumvallate taste buds. (A). SNAP-25+ cells (magenta) in taste buds after saline injections (Saline, top row), a single injection of 75 mg/kg CYP (1CYP, middle row), or five injections of 15 mg/kg CYP (5CYP, bottom row) at 2 days (left column), 6 days (middle column), and 10 days (right column) post injection. Tissues were counter-reacted with Sytox green, a nuclear marker. For 1CYP mice, the number of SNAP-25+ cells were lower 8 and 10 days after injection. For the 5 CYP mice, SNAP-25+ labeling was suppressed 6–10 days post injection. Scale bar = 20 μm. (B) Means ± SEM of SNAP-25+ cells (top graph), total cells (middle graph), and percent of SNAP-25+ cells per taste bud (bottom graph) are plotted over days post injection. For 1CYP mice, SNAP-25+ signal, total cell number, and percent labelled cells were significantly lower than saline control mice at 8 and 10 days after CYP injection. For 5CYP mice, SNAP-25+ and total number of cells per taste bud were lower than saline controls 2–10 days post injection and lower than 1CYP mice at 2–6 days post injection. The percent of SNAP-25+ labeled cells per taste bud in 5CYP mice was higher than 1CYP mice at 8 days and lower than controls at 10 days after injection. All Ps < .05 or less: (1) * 5CYP < Saline, (2) # 1CYP < Saline, and (3) @ 5CYP < 1CYP.

In general, the results of experiment 1 indicated that both CYP dosing regimens disrupted the taste system, although dose fractionation appeared to have a more severe impact on taste buds than a large dose administered in a single injection. Disruptions of cell proliferation lasted longer after multiple small doses than after a single large dose. Dose fractionation of CYP also resulted in a greater loss of cells along with greater loss of differentiated cell types within circumvallate taste buds than seen after a single large dose of CYP (Figs 1–3).

#### Cell proliferation

The ANOVA analysis of the Ki67 data (percent of Ki67+ cells/total cells) indicated significant main effects for dosing conditions [F(2, 73) = 39.056, P<0.001] and days [F(7, 73) = 18.376, P<0.001] as well as for the interaction between the two factors [F(14, 73) = 5.899; P<0.001; see Fig 1]. Simple effects tests and Sidak corrected t-tests found that both CYP dosing methods significantly reduced cell proliferation during the four days immediately after the last injection (Ps<0.001; Fig 1B). However, 1CYP mice showed a sharp increase in the number of proliferating cells between 4 and 6 days post injection, reaching the same rate as SAL mice on day 6. Proliferation levels in 5CYP mice remained suppressed through day 6 (P<0.001) compared to mice in the other two treatment conditions. By day 8, the percentage of proliferating cells of 5CYP mice appeared to be increasing, although this percentage was still significantly lower than the other two treatment conditions (P<0.05). By day 10, proliferation in the 5CYP mice had reached the same level as the mice in the other two dosing conditions.

#### PLCβ2 labeling

Dose fractionation appeared to have a more sustained effect on PLCβ2+ cells and the total number of cells of a circumvallate taste bud than single dosing. The 2-way ANOVA of PLCβ2+ cells found significant main effects for dosing condition [F(2, 65) = 54.293, P<0.001] and day [F(7, 65) = 5.770, P<0.001] factors (Fig 2). It also found a significant interaction between the two factors [F(14, 65) = 3.886, P<0.001]. Further breakdown of these effects indicated that the 5CYP mice had significantly fewer PLCβ2+ cells per taste bud than SAL mice between days 2–12 (days 2–10, P<0.01; day 12, P<0.05; Fig 2B). They also had fewer labeled cells than the 1CYP mice for the first 4 days post injection (P<0.05) and again on day 12 (P<0.01). On the other hand, 1CYP mice had significantly lower levels of PLCβ2+ cells per taste bud than SAL mice on days 8 and 10 (P<0.001).

To determine if either CYP dosing regimen altered the cellular population of these taste buds, the counts of the total number of cell nuclei per taste bud were also analyzed. Significant main effects for dosing condition [F(2, 65) = 25.519, P<0.001] and days [F(7, 65) = 2.193, P<0.05], and their interaction [F(14, 65) = 3.021, P<0.001], were identified (Fig 2B). Post hoc analysis of these data indicated that, compared to SAL mice, the 5CYP treatment reduced the total number of cells per taste bud for 12 days post injection (days 2–10, Ps<0.01; day 12, P<0.05). They also had fewer total cells compared to the 1CYP mice on days 2–4 (Ps<0.05). The 1CYP mice also had significantly fewer total cells per taste bud than SAL mice on days 8 and 10 (Ps<0.05 and 0.01, respectively). Since there was a reduction in the total cellular populations in the circumvallate taste buds, we also examined the percentage of PLCβ2+ cells of the total cell population in these taste buds (Fig 2B). This ANOVA found significant main effects for the two factors (dosing condition [F(2, 65) = 21.904, P<0.001]; days, [F(7, 65) = 5.013, P<0.001]). Post hoc evaluation of these data showed that the 1CYP mice had significantly smaller percentage of PLCβ2+ cells per taste bud than SAL mice on days 8 and 10 (P<0.01 and 0.05, respectively). The 5CYP mice had significantly lower percentages of labeled cells compared to SAL mice on days 6, 8 and 10 (Ps<0.01) and lower than 1CYP mice on day 6 (P<0.01), indicating that the loss of Type II cells was due to more than just a reduction of cells within taste buds.

#### SNAP-25 labeling

Type III cells, identified by labeling of SNAP-25, were also adversely affected by both dosing regimens, although not quite in the same manner, 8–10 days after treatment. The ANOVA of counts of SNAP-25+ cells also detected significant effects of dosing condition [F(2, 65) = 40.017, P<0.001], days [F(7, 65) = 16.016, P<0.001], and their interaction [F(14, 65) = 4.867, P<0.001; Fig 3]. The 5CYP mice had significantly fewer labeled cells for the first 10 days after the last injection (all Ps<0.001) and significantly fewer labeled cells than the 1CYP mice for the first six days (P≤0.05; Fig 3B). The number of SNAP-25+ cells of 1CYP mice decreased on days 8 and 10 to levels significantly lower than SAL mice. The analyses of the total number of cells within these taste buds also detected significant group differences due to dosing condition [F(2, 65) = 36.805, P<0.001] and days [F(7, 14) = 4.277, P<0.001; Fig 3B]. While the 1CYP mice had significantly fewer total cells within taste buds compared to SAL mice on days 8 and 10 (Ps<0.05), the 5CYP mice had significantly fewer total cells than saline mice on days 2–12 (days 2–10, Ps<0.001; day 12, P<0.05). The 5CYP mice also had fewer cells than 1CYP mice on days 4 (P<0.001) and 6 (P<0.05). The analysis of the SNAP-25+ percentage data identified effects of days [F(7, 65) = 4.752, P<0.001] and the interaction of dosing condition by days [F(14, 65) = 2.822, P<0.005; Fig 3B]. The percentage of SNAP-25 cells per taste bud in the 1CYP mice was significantly lower than SAL mice on both days and lower than 5CYP mice on day 8. However, the percentages of SNAP-25 cells of the 5CYP mice were significantly lower than SAL mice on day 10.

In general, fractionated dosing prolonged the disruption of cell proliferation compared to a single large dose of CYP. The large single dose of CYP decreased Type II and Type III cell types primarily 8–10 days post injection. Fractionated dosing tended to increase the time span during which these cells were diminished. Fractionated dosing also caused a larger, more immediate loss of the total number of cells within taste buds than a single dose of CYP. These effects appeared to have been reversed by 16 days post injection.

### Experiment 2: AMF Pre-treatment

**Fig 4:**
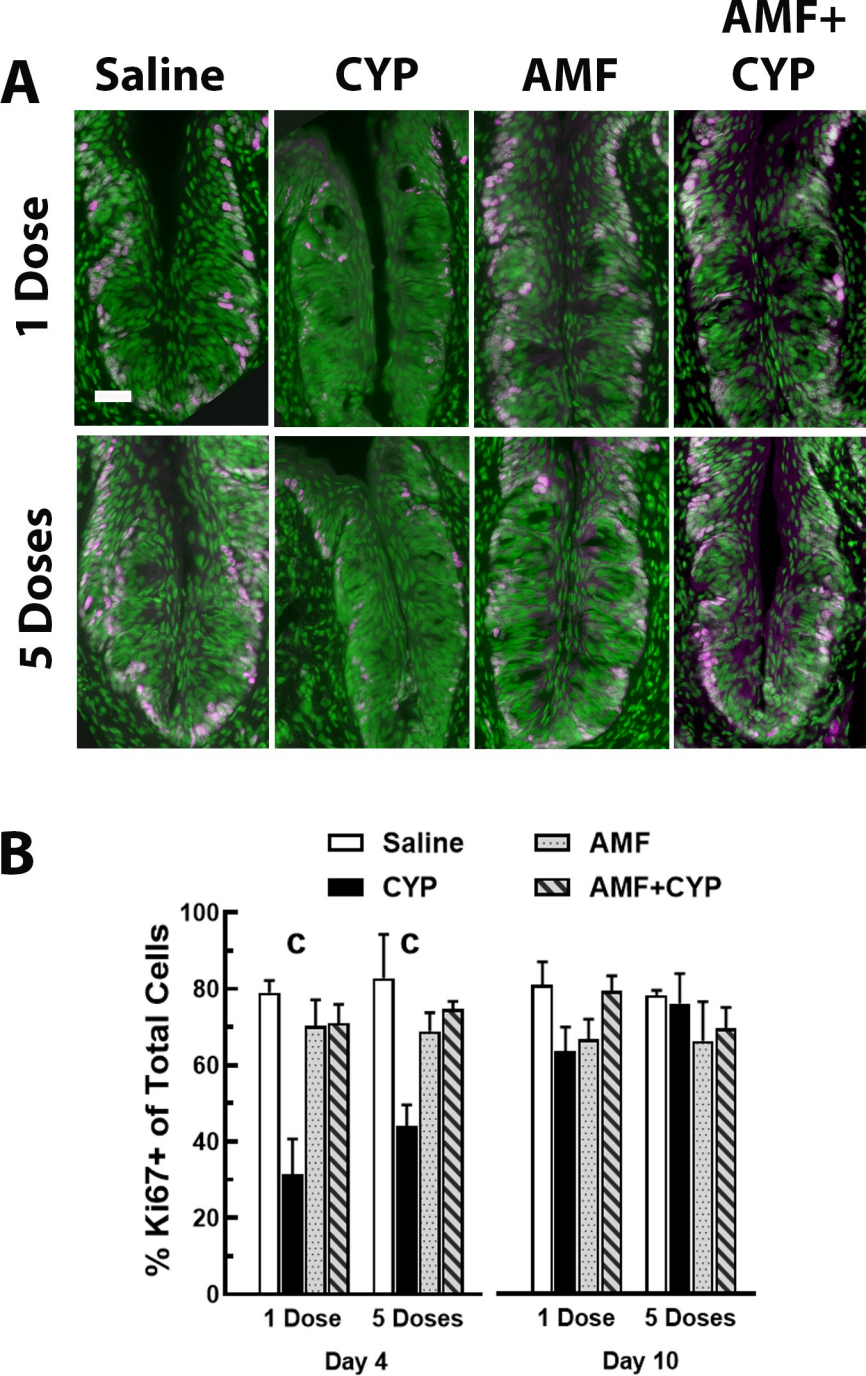
AMF pretreatment protected proliferating cells in the basal layer from the effects of both CYP dosing regimens. (A). Ki67+ cells (magenta) in circumvallate papillae after injections of saline (Saline, column 1 [left]), CYP (column 2), AMF (column 3) or AMF+CYP (column 4) at 4 days post injection. The top row images are examples of Ki67+ cells after single injection conditions and the bottom row images illustrate Ki67+ cells after five injections (fractionated dosing). Tissues were counter-reacted with Sytox green, a nuclear marker. For 1CYP and 5CYP mice, Ki67+ cells were nearly absent compared to all other conditions. Scale bar = 20 μm. (B) Means ± SEM percent of Ki67+ cells relative to total cells in each drug condition at 4 (left panel) and 10 days (right panel) post injection. Both CYP dosing methods reduced Ki67 expression at 4 days but not 10 days post injection. AMF appeared to protect cell proliferation at both time points. Neither AMF dosing method appeared to alter Ki67 expression. All Ps < .05 or less: (1) * 5CYP < Saline, (2) # 1CYP < Saline, and (3) @ 5CYP < 1CYP.

**Fig 5:**
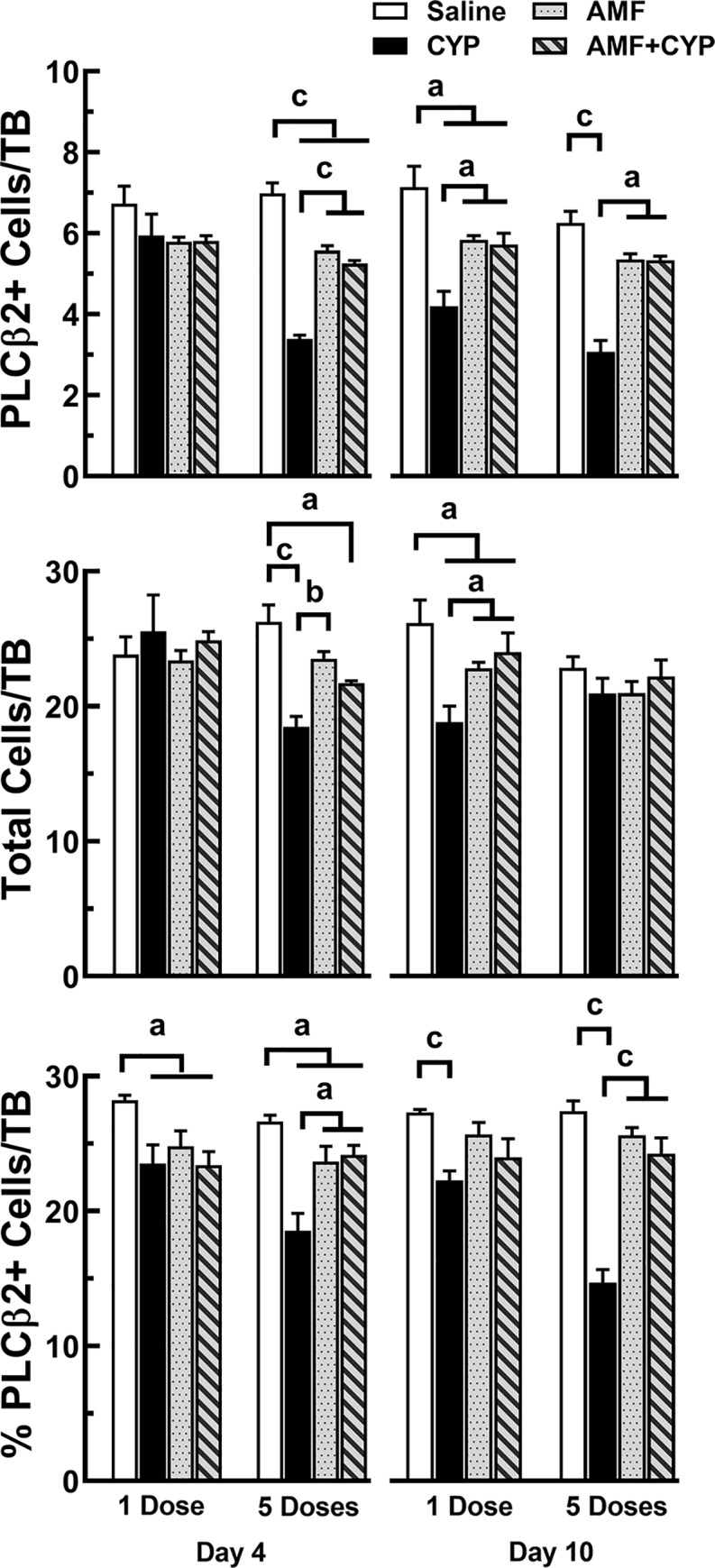
AMF pretreatment protected PLCβ2-expressing cells in taste buds of circumvallate papillae from CYP. Means + SEM of PLCβ2+ cells (top graph), total cells in taste buds (middle graph), and percent of PLCβ2+ cells/taste bud (bottom graph) over days post injection. Day 4 data are shown in the left half and day 10 day are shown the right half of each graph. The bars (left to right) show means (+SEM) for saline, CYP, AMF and AMF+CYP drug groups of mice given their drug treatment either as a single dose or five doses. CYP injections reduced PLCβ2+ signal and total cells per taste bud at day 10 for those mice receiving only one inject of the drug. However, mice receiving 5 injections of CYP showed reduced values for all three measures on both days. All measures of AMF+CYP mice indicated that AMF protected these mice from the effects of CYP. On the other hand, PLCβ2 measures for mice receiving AMF were sometimes lower than the PLCβ2 measures for saline mice. a = P<0.05; b = P<0.01, c = P<0.005.

**Fig 6:**
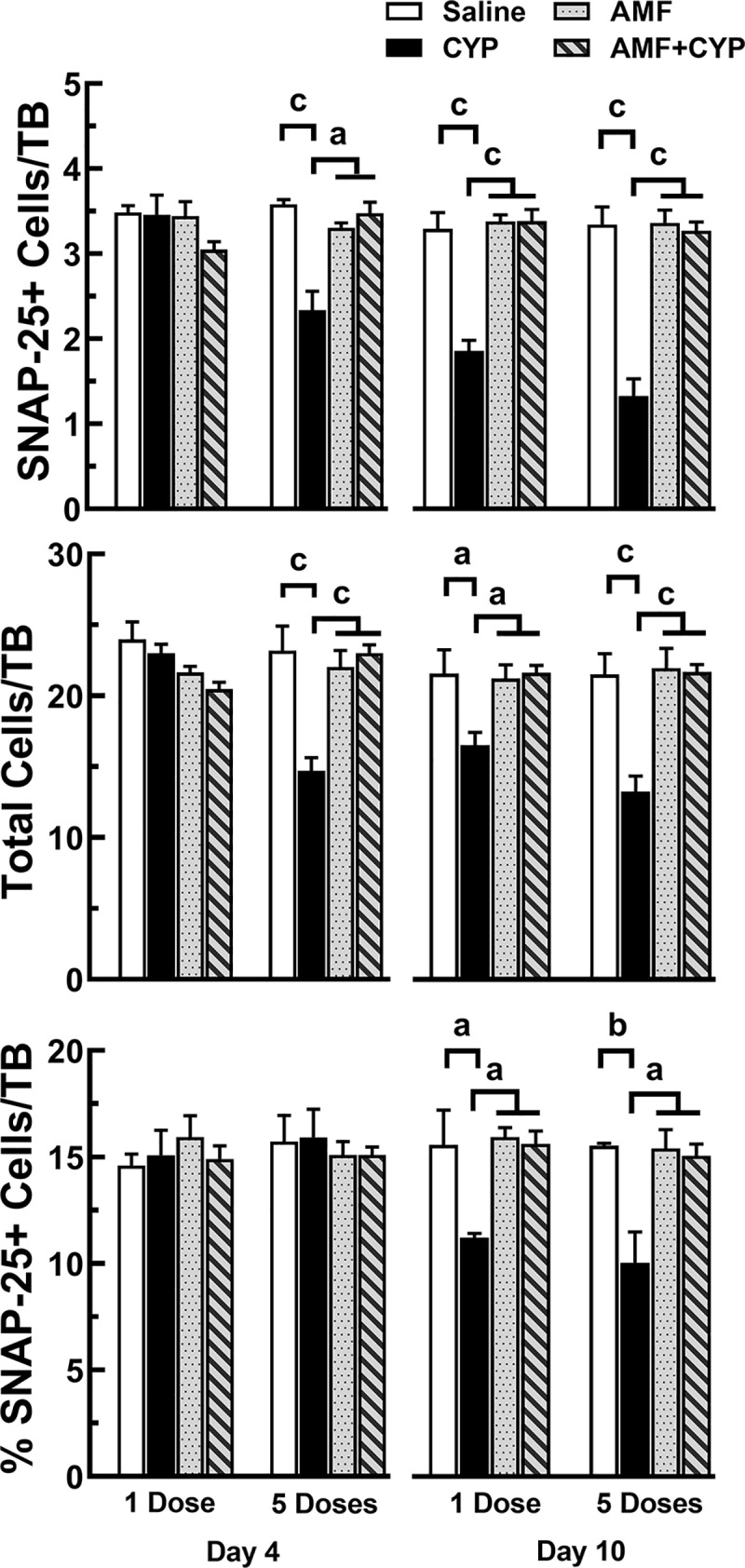
AMF pretreatment protected SNAP-25+ cells in taste buds of circumvallate papillae from CYP. Means + SEM of SNAP-25+ cells (top graph), total cells in taste buds (middle graph), and percent of SNAP-25+ cells/taste bud (bottom graph) over days post injection. Day 4 data are shown in the left half and day 10 data are shown in the right half of each graph. The bars (left to right) show means (+SEM) for saline, CYP, AMF and AMF+CYP drug groups for mice given their drug treatment either as a single dose or five doses. CYP injections reduced SNAP-25+ signal, total cell, and percent per taste bud measures at day 10 for mice receiving one inject of CYP. However, mice receiving 5 injections of CYP showed lower values for all three measures on 10 day and all but percentage measures on day 4. All measures of AMF+CYP mice indicated that AMF protected these mice from the effects of CYP. SNAP-25+ in mice receiving AMF appeared unaffected by either dosing method. a = P<0.05; b = P<0.01, c = P<0.005.

Experiment 2 was designed to test two questions about AMF: 1) would AMF protect the taste system from fractionated dosing with CYP, and 2) would repeated exposure to AMF have detrimental effects on the taste system when presented by itself or in combination with CYP? Because the first experiment revealed effects of CYP on cell proliferation and on individual cell types as well as the overall cellular population within taste buds, all three cellular measures were examined in this experiment at 4 and 10 days after drug treatment (Figs 4–6).

#### Cell proliferation

The Ki67 data were initially evaluated with a 3-way ANOVA to examine the effects of day, dosing, and drug treatment factors. This ANOVA detected significant effects for day [F(1, 42) = 11.075, P<0.001], drug treatment [F(3, 42) = 17.614, P<0.005], and the interaction between day and drug treatment [F(3, 42) = 11.116, P<0.001]. A breakdown of the interaction showed that the 1CYP and 5CYP conditions on day 4 reduced the percent of Ki67+ cells per taste bud significantly lower than all other treatment groups on either day (Fig 4).

#### PLCβ2 cells

As seen in the first experiment, Type II cells were affected more by dose fractionation than single dosing, but AMF was able to protect taste cells from either CYP dosing regimen without apparent adverse effects. The 3-way ANOVA of the PLCβ2 cell counts per taste bud detected significant main effects for day [F(1, 42) = 4.092, P<0.05], dosing [F(1, 42) = 22.799, P<0.001], and drug treatment [F(3, 42) = 54.058, P<0.001] as well as significant dosing by drug [F(1, 42) = 6.104, P<0.005] and day by dosing by drug treatment [F(3, 42) = 3.370, P<0.05] interactions (Fig 5, top graph). These data were then partitioned by the day factor for further analysis. For the day 4 data, no significant group differences in PLCβ2+ cells per taste bud were found for mice given a single dosing but group differences were detected for mice receiving 5 doses of their drug. Post hoc testing with Sidak corrections indicated that the 5SAL mice had the highest counts, which were significantly greater than the other three dose fractionated treatment groups (5AMF or 5AMF+CYP, P<0.01; 5CYP, P<0.005). In addition, the number of labeled cells per taste bud in the 5CYP mice were significantly lower than 5AMF and 5AMF+CYP mice (Ps<0.005). At 10 days post treatment, PLCβ2 labeled cells of 1SAL mice not only were significantly more than the 1CYP (P<0.005) but also exceeded those of the 1AMF and 1AMF+CYP mice (Ps<0.05). The two groups pretreated with AMF also had more PLCβ2+ cells per taste bud than the 1CYP mice (Ps<0.005). PLCβ2 labeling in the fractionated dosing groups were similar. Labeling in 5SAL mice was significantly greater than the 5CYP mice (P<0.005) but not the 5AMF or the 5AMF+CYP mice. However, the number of PLCβ2+ cells per taste bud in the 5AMF and 5AMF+CYP mice were significantly greater than labeling in the 5CYP mice (Ps<0.005).

The total number of cells per taste bud also were affected by dosing [F(1, 42) = 6.308, P<0.05], drug [F(3, 42) = 7.056, P<0.005] and the interaction between days, dosing, and drug conditions [F(3, 42) = 7.123, P<0.005]. Partitioning these data by days post injection revealed significant group differences in the fractionated dosing condition 4 days post injection and in the single dosing condition 10 days post injection (Fig 5, middle graph). The SAL mice had significantly more total cells per taste bud than all other groups (Ps<0.05 or less) in their corresponding dosing conditions. At day 4, the 5AMF group had significantly more cells per taste bud than the 5CYP group. The mean number of cells per taste bud of the 5AMF+CYP group was higher than the 5CYP mice but did not reach significance (P = 0.062). However, at day 10, the 1AMF and 1AMF+CYP groups had significantly more total cells per taste bud than the 1CYP group (Ps<0.05).

The analyses of the percent of PLCβ2+ cells per taste bud revealed significant effects of dosing [F(1, 42) = 13.781, P<0.001], drug treatment [F(3, 42) = 48.770, P<0.001], and the interaction of dosing by drug treatment [F(3, 42) = 10.826, P<0.001]. Post hoc evaluations showed that 1SAL and the 5SAL mice had significantly larger percentages of labeled cells per taste bud on day 4 than the other three drug treatment conditions (Ps<0.05 or less; Fig 5, bottom graph). Of the two CYP groups, however, only the 5CYP mice had significantly lower percentages of PLCβ2+ cells than the other three fractionated dosing groups (Ps<0.05 or less). By day 10, of the single injection groups, only 1SAL mice had significantly higher percentages of PLCβ2+ cells per taste bud than 1CYP mice (P<0.005). On the other hand, 5SAL, 5AMF and the 5AMF+CYP mice had significantly higher percentages of PLCβ2+ cells per taste bud than the 5CYP group (all Ps<0.005).

#### SNAP-25 cells

Type III cells were also affected more by CYP dose fractionation than single dosing, and AMF protected taste cells from the effects of both regimens without apparent adverse effects. The 3-way ANOVA of the SNAP-25+ cell data identified significant main effects of day [F(1, 47) = 22.857, P<0.005], dosing [F(1, 47) = 4.826, P<0.05] and drug treatment [F(3, 47) = 55.008, P<0.005], and significant interactions of day by drug treatment [F(3, 47) = 17.532, P<0.005] and dosing by drug treatment [F(3, 47) = 8.763, P<0.005]. At 4 days post injection, mice receiving single doses of these drugs did not differ from 1SAL mice. On the other hand, the 5SAL, 5AMF, and 5AMF+CYP groups had significantly more labeled cells per taste bud than the 5CYP mice (all Ps<0.005; Fig 6, top graph). At 10 days post injection, the SNAP-25+ cell data showed that for both single-injection and five-injection conditions, the SAL mice had significantly more labeled cells per taste bud than the CYP groups in their respective dosing condition (Ps<0.005). Likewise, the AMF and AMF+CYP groups in each dosing condition had significantly more labeled cells than their respective CYP mice (Ps<0.005).

The analysis of the total number of cells per taste bud found significant effects of day [F(1, 47) = 8.454, P<0.01], drug treatment [F(3, 47) = 23.102, P<0.005], and day by drug treatment [F(3, 47) = 8.731, P<0.005]. Further analysis of the day 4 and day 10 data found significantly fewer cells per taste bud in the 1CYP and 5CYP mice than the other groups (Ps<0.005; Fig 6, middle graph) in their respective days post injection. In addition, the number of cells per taste bud of 1CYP mice were also significantly lower than the 1SAL, 1AMF or the 1AMF+CYP mice (Ps<0.05).

The ANOVA of the percent of SNAP-25+ cells per taste bud also indicated significant day [F(1, 47) = 4.480, P<0.05], drug treatment [F(3, 47) = 6.483, P<0.005], and day by drug treatment [F(3, 47) = 7.747, P<0.005] effects. Post hoc analysis of the data showed no effect of either dosing regimen 4 days post injection. However, by 10 days post injection, 1CYP and 5CYP mice had significantly lower percentages than the other three groups in their respective dosing condition (all Ps<0.05; Fig 6, bottom graph).

In general, the effects of 1CYP and 5CYP treatment on PLCβ2 and SNAP-25 cells were similar to findings (cell proliferation, labeled cell counts, total cells, and percentage of cells/taste bud) of experiment 1. In addition, AMF appeared to protect the cells of the taste system from the effects of both CYP dosing regimens. Although AMF did not always preserve all cell counts at the same level as measured in control mice, AMF alone did not appear to have major detrimental effects from repeated administration.

## Discussion

The findings of this study provide new insights into how the taste system may be impacted by different dosing regimens a patient might encounter during chemotherapy. They also give us new insights into the temporal patterns of the effects of CYP on cellular losses and cell renewal in the taste system. One of the most important insights is how important the dosing regimen is for cell proliferation and cell renewal. Both CYP dosing regimens effectively suppressed cell cycling to about the same level immediately after the last injection of CYP. Ki67 expression in mice given a single large dose of CYP remained suppressed up to four days post injection but then appeared to increase to control levels by the sixth day. In contrast, Ki67 expression in mice given CYP by dose fractionation remained suppressed much longer. After fractionated dosing of CYP, Ki67 expression began to increase after six days but more gradually, eventually reaching control levels 10 days after the last injection. While neither dosing regimens stopped cell proliferation completely, both were able to slow this process down to the point where cell renewal did not appear to keep up with the loss of cells when they reached the end of their life span.

The differences between the two dosing regimens can also be extended to the cellular populations within taste buds. After a single injection of CYP, the population of PLCβ2+ cells began to decrease 6 days after injection, reaching its low point on days 8 and 10 before returning to control levels 12 days post injection, findings comparable to previous studies [16, 17, 19]. This period coincided with a decrease in the total number of cells within taste buds, but the decrease in the percent of PLCβ2+ cells during this same period suggests that the loss of Type II cells exceeded the overall rate of cell loss. On the other hand, fractionating the dose of CYP enhanced the disruptive effects of the drug on Type II cells. Mice given the small doses of CYP over five consecutive days had fewer PLCβ2+ cells per taste bud than either of the other two conditions 2 days after the last injection of CYP, which was also 7 days after the beginning of the dosing regimen. The number of PLCβ2+ cells remained lower than control levels for 12 days after the last injection before returning to control levels. This pattern also corresponded to the total loss of cells from the taste buds of the 5CYP mice but again the percent of PLCβ2+ cells within taste buds dropped more between days 6–10 than the rate of general cell loss. While fractionated dosing of CYP caused a greater disruption of PLCβ2+ cells in taste buds than a single dose, the timing of maximum loss of Type II cells caused by both regimens corresponds to the anticipated end of the life spans of Type II cells surviving the cytotoxic effects of CYP, but without adequate cell renewal to replace them.

Similar temporal patterns of cellular loss were observed for SNAP-25+ cells in each dosing condition, with one major point of deviation. Like an earlier study [19], the percent of SNAP-25+ cells in circumvallate taste buds of 1CYP mice showed a sharp drop at day 8, which started to reverse by day 10, and returned to control levels by day 12 post drug treatment. The percent of SNAP-25+ cells of 5CYP mice indicates a similar magnitude of loss in Type III cells but the onset of this loss was delayed by at least two days. This decrease in Type III cells did not last as long as the effects of a single injection, since these cells also returned to control levels 12 days post drug treatment. Potential explanations for these losses are not fully apparent since the half-life of Type III cells are now estimated to be 22–24 days [30]. Instead, these data suggest that the loss of Type III cells may be related to other events within taste buds. One possible explanation, for example, may be based on the coincidental decrease in the rate of cell renewal and the drop in the number of Type II cells in 1CYP mice that preceded the loss of Type III cells. One might speculate that the reduction of Type II cells to some critical level also reduced cell-cell signal interactions needed to maintain Type III cells within a taste bud. However, this explanation is not supported by the data for 5CYP mice since the percentage of PLCβ2 cells in their taste buds dropped to its lowest level 6 days post injection and the spike-like drop in Type III cells did not occur until 10 days post treatment. A second possibility is that a sharp decrease in Type III cell population occurred after a critical decrease in the population of Type I cells. Most Type I cells have a half-life of 6–8 days [30] and are known to provide glial-like functions that may be critical for the survival of Type III taste sensory cells after CYP treatment. It is also possible that a loss of Type I cells could contribute to the decrease in Type II cells. Type I cells were not included in these experiments because their morphological structure entwines them around multiple cells within a taste bud [20], making it difficult to quantify these cells accurately using the methods employed in this study [51]. However, dose fractionation of CYP produced more prolonged and more cyclic increases in salt thresholds of mice, an important behavioral function of Type I cells which would be disrupted with their loss [37]. This hypothesis can be extended further if one assumes that after CYP treatment and the cell renewal process is restarted, cell differentiation may initially be biased toward replacing Type I cells to prepare the taste bud environment before differentiation and maturation of Type II or Type III cells. Since dose fractionation depressed cell proliferation for a much longer time than single dosing, it may have altered the rate at which each cell type was renewed. If so, then one would expect the components of Wnt/β-catenin pathway, Sonic Hedgehog, Sox2 and other molecular signals [31–35, 53–56] that contribute to the regulation of cell proliferation and maturation of taste cells after catastrophic insult, such as seen with CYP, would adjust in ways necessary to reinstate the functionality of the taste system. The differential results of dosing seen in this study suggest that the interconnected responses of these regulatory mechanisms may vary in ways driven by how the population of cells in the taste bud is damaged. A third possibility derives from CYP’s ability to induce an inflammatory reaction in epithelial tissues, including the bladder and the tongue [48–50, 57–59]. Feng et al. have shown that a subset of Type II cells that express T1R3, also express the proinflammatory agent, TNF-α, which can induce inflammation in other taste cells, shorten their life spans, and interrupt cell proliferation [57, 60–62]. On the other hand, Type III cells produce the anti-inflammatory agent, IFN-γ, that may counter or downregulate an inflammatory reaction [63, 64]. Perhaps the T1R3 subset of Type II cells is more sensitive to the inflammatory reaction induced by CYP, especially if repeated dosing of CYP activates a stronger, cumulative inflammatory reaction than a single dose, whereas Type III cells are partially protected against that inflammatory response. Tests of these or other hypotheses and how dosing regimen might affect these processes will require further research. The differences in the timing of the rapid decrease in Type III cells related to the dosing regimen may provide clues to solving the reason for post injection Type III cell losses.

AMF appeared to prevent CYP-induced loss of cells within taste buds and protect the cells involved in taste cell renewal. While both dosing regimens suppressed the level of Ki67+ cells 4 days after the last drug treatment, the levels of Ki67 expression in mice treated with AMF alone or given as a pretreatment before CYP were comparable to saline control mice. Similar findings were observed for levels of SNAP-25+ cells. AMF also prevented the loss of PLCβ2+ cells, although like a previous report [16, 17], not as effectively (Fig 5–6), especially when AMF was the pretreatment for the higher, single dose of CYP. This dosing regimen may require a higher dose of AMF for more complete protection. It is also possible that AMF may protect Type I cells like it does Types II and III cells. Perhaps Type III cells, which are responsive to environmental pH levels, can absorb AMF more effectively than Type II cells. It should be noted that repeated dosing with AMF (100 mg/kg) for 5 consecutive days appeared to have some impact on the total number of cells and the number of Type II cells per taste bud, but this appeared to be much less than the effects of either CYP dosing regimen and clearly did not prevent the drug from protecting the taste system from the toxic effects of CYP.

These findings agree well with those reported for AMF (100 mg/kg) and CYP (single injection of 75 mg/kg) by Mukherjee et al. [16, 17]. In that study, the cellular effects of AMF and CYP were examined 4, 7, 10 and 16 days post injection and behavioral thresholds for sucrose were measured daily. AMF alone had little effect on sucrose thresholds of mice but reduced the amount and duration of the CYP-induced 2-phase elevations in thresholds after CYP administration. In CYP mice the number of fungiform papillae dropped by 50% by day 4 post injection and did not recover until day 16. AMF alone caused a loss of about 25% of these papillae but protected the remainder from the toxic effects of CYP. Like the current study, pretreatment with AMF protected cell proliferation associated with fungiform and circumvallate taste buds from disruption by CYP while having no effect on proliferation when injected by itself. In addition, immunolabeling of PLCβ2+ cells in circumvallate taste buds was decreased by CYP treatment 7 and 10 days post injection but AMF prevented this loss on day 7 and reduced the loss on day 10 post injection. PLCβ2+ cells in fungiform taste buds showed a significant loss on days 4, 7, and 10 but pretreatment with AMF limited Type II cell loss in fungiform taste buds to day 7. AMF by itself did not affect Type II cells at any time point during the experiment. Three points emerged from the Mukherjee et al. study: 1) fungiform taste fuds were more sensitive to the cytotoxic effects of CYP (also observed in another study [18]), 2) AMF protected the peripheral taste system from the effects of CYP, and 3) AMF alone had some mild negative effects on the taste system. The present study extended these findings to Type III cells in circumvallate taste buds and to the effects of different dosing regimens. While fungiform taste buds were not examined systematically in this study, the comparable findings of the two studies suggest that fungiform taste buds could be more susceptible to the fractionated dosing regimen (see also S1 Fig for images of fungiform taste buds after each dosing regimen). In addition, while AMF may also have a mild negative effect on fungiform taste buds, pretreatment with AMF is likely to be as effective at protecting fungiform taste buds from the effects of CYP as it was with circumvallate taste buds in this study. Further research is needed to evaluate this hypothesis and to determine how AMF protects taste sensory cells.

In summary, fractionated dosing of CYP appeared to prolong the effects of CYP on cell renewal of the peripheral taste system. In addition, it enhanced the duration of total cell and Type II cell losses within taste buds. The timing of these losses is related to the life span of Type I and II cells. Type III cells were also susceptible to the effects of either dosing regimen, but the loss of these cells may be due to factors related to the general health and/or cell renewal of taste buds rather than the life span of Type III cells. Further research is needed to determine how CYP affects the population of Type I and Type III cells. In general, pretreatment with AMF appeared to protect taste cell renewal and the population of cells within taste buds from the cytotoxic effects of CYP. These findings may have important clinical implications for patients subjected to chemotherapy [38–40].

## Supporting information

S1 Fig PLCβ2 expressed in fungiform papillae 4 days post injection was reduced by the two CYP dosing regimens. PLCβ2+ cells (magenta) in fungiform papillae 4 days after (A) saline injection, (B) a single injection of 75 mg/kg CYP, or (C) five injections of 15 mg/kg CYP. Tissues were counter-reacted with Sytox green, a nuclear marker. Both dosing regimens decreased the number of PLCβ2+ cells compared to saline injected control mice. Scale bar = 20 μm.
(TIF)

S1 Table Summary of the number of mice evaluated for each immune-positive marker in each of the four drug treatments and three dosing conditions (SAL, 1CYP and 5CYP) across days post injection in experiment 1. (DOCX)

S2 Table Summary of the number of mice evaluated for each immune-positive marker in each of the two dosing conditions and four drug treatments 4 or 10 days post injection in experiment 2. (DOCX)
