# Supplementary figures and images for: Cyclophosphamide and the taste system: Effects of dose fractionation and amifostine on taste cell renewal
Source: PLoS One. 2019 Apr 4;14(4):e0214890. doi: 10.1371/journal.pone.0214890 (PMC6448888; doi:10.1371/journal.pone.0214890)

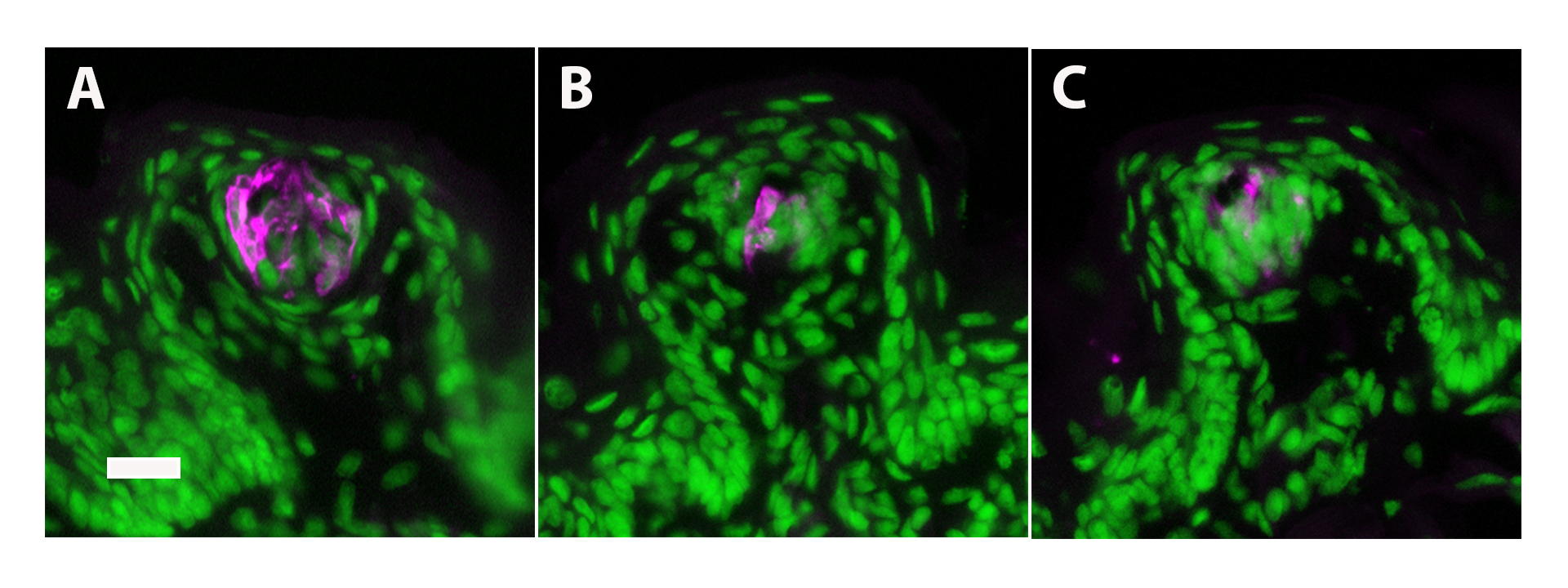

Supplement: S1 Fig — PLCβ2+ cells (magenta) in fungiform papillae 4 days after (A) saline injection, (B) a single injection of 75 mg/kg CYP, or (C) five injections of 15 mg/kg CYP. Tissues were counter-reacted with Sytox green, a nuclear marker. Both dosing regimens decreased the number of PLCβ2+ cells compared to saline injected control mice. Scale bar = 20 μm. (TIF) [file pone.0214890.s001.tif]
