# Supplementary material for: Cyclophosphamide and the taste system: Effects of dose fractionation and amifostine on taste cell renewal
Source: PLoS One. 2019 Apr 4;14(4):e0214890. doi: 10.1371/journal.pone.0214890 (PMC6448888; doi:10.1371/journal.pone.0214890)
Supplement: S1 Table — (DOCX) [file pone.0214890.s002.docx]

**S1 Table. Summary of the number of mice evaluated for each immune-positive marker in each of the four drug treatments and three dosing conditions (SAL, 1CYP and 5CYP) across days post injection in experiment 1.**

**Ki67**

**Days Post Injection**

| **Dose Regimen** | **2** | **4** | **6** | **8** | **10** | **12** | **14** | **16** |
| --- | --- | --- | --- | --- | --- | --- | --- | --- |
| **SAL** | 4 | 4 | 3 | 6 | 5 | 4 | 5 | 5 |
| **1CYP** | 4 | 4 | 6 | 6 | 5 | 4 | 4 | 3 |
| **5CYP** | 4 | 4 | 4 | 4 | 4 | 4 | 3 | 4 |

**PLCβ2**

**Days Post Injection**

| **Dose Regimen** | **2** | **4** | **6** | **8** | **10** | **12** | **14** | **16** |
| --- | --- | --- | --- | --- | --- | --- | --- | --- |
| **SAL** | 3 | 5 | 3 | 3 | 4 | 3 | 3 | 3 |
| **1CYP** | 5 | 4 | 4 | 4 | 4 | 5 | 3 | 4 |
| **5CYP** | 5 | 5 | 4 | 4 | 4 | 3 | 3 | 3 |

**SNAP-25**

**Days Post Injection**

| **Dose Regimen** | **2** | **4** | **6** | **8** | **10** | **12** | **14** | **16** |
| --- | --- | --- | --- | --- | --- | --- | --- | --- |
| **SAL** | 4 | 3 | 4 | 4 | 4 | 3 | 4 | 3 |
| **1CYP** | 3 | 4 | 4 | 4 | 4 | 3 | 4 | 3 |
| **5CYP** | 4 | 4 | 4 | 4 | 4 | 3 | 4 | 4 |
