# Supplementary material for: Cyclophosphamide and the taste system: Effects of dose fractionation and amifostine on taste cell renewal
Source: PLoS One. 2019 Apr 4;14(4):e0214890. doi: 10.1371/journal.pone.0214890 (PMC6448888; doi:10.1371/journal.pone.0214890)
Supplement: S2 Table — (DOCX) [file pone.0214890.s003.docx]

**S2 Table. Summary of the number of mice evaluated for each immune-positive marker in each of the two dosing conditions and four drug treatments 4 or 10 days post injection in experiment 2.**

**Immuno-positive Marker**

**Ki67 PLCβ2 SNAP-25**

**Dose Drug Days post injection**

**4 10 4 10 4 10**

| **1 Dose** | **SAL** |  | 4 | 4 |  | 4 | 4 |  | 4 | 4 |
| --- | --- | --- | --- | --- | --- | --- | --- | --- | --- | --- |
|  | **CYP** |  | 4 | 4 |  | 4 | 3 |  | 4 | 3 |
|  | **AMF** |  | 3 | 3 |  | 3 | 3 |  | 4 | 4 |
|  | **AMF+CYP** |  | 3 | 3 |  | 3 | 3 |  | 4 | 4 |
|  |  |  |  |  |  |  |  |  |  |  |
| **5 Doses** | **SAL** |  | 4 | 4 |  | 4 | 4 |  | 4 | 4 |
|  | **CYP** |  | 4 | 4 |  | 4 | 4 |  | 4 | 4 |
|  | **AMF** |  | 3 | 3 |  | 3 | 4 |  | 4 | 4 |
|  | **AMF+CYP** |  | 4 | 4 |  | 4 | 4 |  | 4 | 4 |
